# The Effect of the COVID-19 Pandemic on Palestinian Patients Attending Selected Governmental Hospitals: An Analysis of Hospital Records

**DOI:** 10.3390/ijerph21020196

**Published:** 2024-02-08

**Authors:** Mousa Atary, Niveen M. E. Abu-Rmeileh

**Affiliations:** Institute of Community and Public Health, Birzeit University, Ramallah P.O. Box 14, Palestine; nrmeileh@birzeit.edu

**Keywords:** COVID-19 pandemic, outpatient clinics, operation deferral, lockdown, shifting of resources, Palestinian Authority

## Abstract

Introduction: Confronting the COVID-19 epidemic forced the closure or relocation of the majority of health facilities. It is likely that non-COVID-19 patients suffered collateral effects. Methods: The clinic and operating room records were analyzed at selected Palestinian government hospitals in the West Bank region. Results: The reduction in patient clinic visits varied from 49% to 90%, with Ear-Nose-Throat (ENT), urology, and pediatric clinics being the most affected. The reduction in operation numbers in the center (which had independent decision-making) ranged from 7.1% to 23.4%, but in the north and south (which followed centralized choices), the reduction ranged from 19.6% to 91.8%. Conclusions: COVID-19 affected outpatient visits. The pandemic affected some services, but West Bank hospitals were able to provide normal obstetric and gynecological treatments and help patients who needed primary or intermediate surgery.

## 1. Introduction

Access to healthcare for non-COVID-19 patients was compromised during the COVID-19 pandemic due to the increase in patient demand and supply concerns. Patients infected with COVID-19 were afraid of it, and administrative orders and the protection of children and the elderly contributed to the surge in demand for health services. The supply variable included a scarcity of personal protective equipment (PPE), medical instruments such as ventilators, qualified healthcare professionals, beds, and ICU beds. The result of these factors was an overall decrease in visits to emergency rooms and a decrease in operations performed [1,2,3,4,5,6].

In Brazil, elective procedures were reduced by 35%; this drop was dynamic in response to pandemic waves [7]. As a result of COVID-19, people’s health, social lives, and economic status were affected [2].

Resource reallocation was carried out to address the shortage of supplies more effectively. Clinics were transformed into inpatient units. Ventilators were relocated from operating rooms to these newly created wards. Operative teams were reassigned to handle the patients in these wards, and PPE was reallocated to COVID-19 management wards [8,9,10,11]. In Palestine, the first COVID-19 instances were recognized on 5 March 2020. On 13 March 2020, a complete lockdown of the West Bank began [12]. The Palestinian Ministry of Health issued a protocol for the reopening of outpatient clinics in May 2020 to enable the reopening of clinics. Nonetheless, most governmental hospitals’ outpatient clinics remained closed until October 2020. In November 2020, a more thorough action plan for dealing with the COVID-19 pandemic was developed, which included the establishment of new facilities to treat COVID-19 patients [13].

The purpose of this research is to ascertain the effects of the COVID-19 pandemic on elective hospital service utilization. We will analyze the association between the COVID-19 epidemic and the change in the number of patients visiting outpatient clinics, the change in the pattern of healthcare utilization, and the change in the number and kind of procedures performed in Palestinian public hospitals. As a result, we anticipate a decrease in the number of cases, operations, and persons seeking health care.

## 2. Materials and Methods

### 2.1. Data Source

This study is based on a secondary analysis of data extracted from the Palestinian Ministry of Health (MoH) hospital records. A governmental hospital is present in each West Bank and Gaza Strip governorate. The largest are the Rafedia, Palestine Medical Complex, and Alia hospitals [14]. Each hospital contains outpatient clinics, inpatient wards (regular or closed (ICU)), operating rooms, and other related support services [14]. The governmental medical insurance in Palestine covers many people; it covers governmental employees, workers in Israel, cancer patients, hematology patients, disabled patients, and those receiving social aid. In addition, it covers the unemployed, children under than three years old, martyr’s families, prisoners in Israel, athletes, union members, and anyone who is willing to participate in it. In March 2020, all COVID-19 patients were added [15]. The chosen three tertiary hospitals in the West Bank were Palestine Medical Complex (PMC) (central in the West Bank), Rafedia Hospital (northern area of the West Bank), and Alia Hospital (southern area of the West Bank). The data from each hospital are described in Table 1. 

### 2.2. Patients and Setting

During the pandemic, three two-week intervals were determined based on the number of COVID-19 cases and preventive actions performed. These intervals represented our study period. The first phase—the first two weeks of May 2020—was in the midst of the initial lockdown, during which information about COVID-19 was scant, suspect, and contradictory. Between the first and second COVID-19 waves in Palestine, the second period encompasses the first two weeks of September 2020. The lifting of the lockdown and the reopening of markets created a sense of security. The third period encompasses the first two weeks of January 2021 and corresponds to the pandemic’s second wave. A deft lockdown was implemented (closing off markets after 7:00 p.m. and on weekends, schools and universities used distance learning, and wedding halls were closed). However, COVID-19 had a high mortality rate (reaching 5.41 percent of cases in the third wave, up from 0.012 percent in the second wave and from 0.02 percent in the first wave) [15,17]. Due to their prior experience with the lockdown’s economic consequences, many felt doubtful and more hesitant to obey.

These periods were compared to the preceding year, 2019, in order to account for seasonal fluctuations such as schools starting, summer vacations, and holidays.

Excel was used to create two data extraction tables for outpatient clinics and operating rooms. The outpatient clinic data table comprised information about the number of visitors, their age, gender, the kind of insurance used at each clinic, and the total number of clinics in each hospital. The operation rooms table included information about the number of operations performed in each hospital during each period, the type of surgery performed, and the gender distribution.

During the allocated six periods, patients who attended outpatient clinics or underwent surgery at the three hospitals analyzed were evaluated. The data set includes all patients, despite the fact that some patients lacked age and gender information. General surgery, neurosurgery, orthopedic surgery, pediatric surgery, vascular and thoracic surgery, ear-nose-throat (ENT), ophthalmology, gynecology and obstetrics, internal medicine, pediatrics, oncology, cardiology, and neurosurgery were conducted in the outpatient clinics.

Between 24 March and 30 August 2021, the Ministry of Health extracted data from its central computerized registry. The data were exported to an Excel spreadsheet.

### 2.3. Variables

The outpatient clinic data were devoid of any identifiable information. Nonetheless, they featured information about the number of patients who attended the clinics, their age (in years-month-days format), gender, clinic type, insurance type, and physician’s name. Additionally, the data from the operating room included a list of patients without their names, their gender, their ages (in years-months-day format), and the type of operation (superlong, long, medium, and minor).

Another set of tables was derived from other hospital sources for data quality checks: physicians’ tables giving the number of patients seen during the same research periods, insurance statistics, and a table containing the gender and age of each clinic patient. They were compared to the actual tables extracted from the Ministry of Health. For all variables, missing data accounted for less than 1% of total cases.

### 2.4. Statistical Analysis

Excel was also used to analyze the data, including each clinic’s average age and gender. The operating room records comprised information about the number of operations performed throughout each period, which was retrieved directly from the Ministry of Health. The reported operations were classed as mild, intermediate, or major in accordance with the NICE criteria [15].

Additionally, using the *t*-test, we compared the pre-and post-COVID-19 periods in each region using IBM SPSS Statistics for Windows, version 25 (IBM Corp., Armonk, NY, USA), Palestine, 2022, setting the significant *p*-value to be less than 0.05.

The effect of the COVID-19 pandemic on patient attendance at clinics, operations performed, and gender distribution was compared using a *t*-test for each region. The significance level was considered when the *p*-value < 0.05

## 3. Results

The pandemic era saw 2237, 5470, and 3938 patients at the north, center, and south clinics, respectively; the pre-pandemic period saw 7811, 10,743, and 11,227 people.

A *t*-test revealed a statistically significant difference (decrease in the pandemic) in the number of patients seen in clinics in the north (Rafedia) and south (Alia) but not in the center (PMC), as shown in Table 2. 

**Figure 1:**
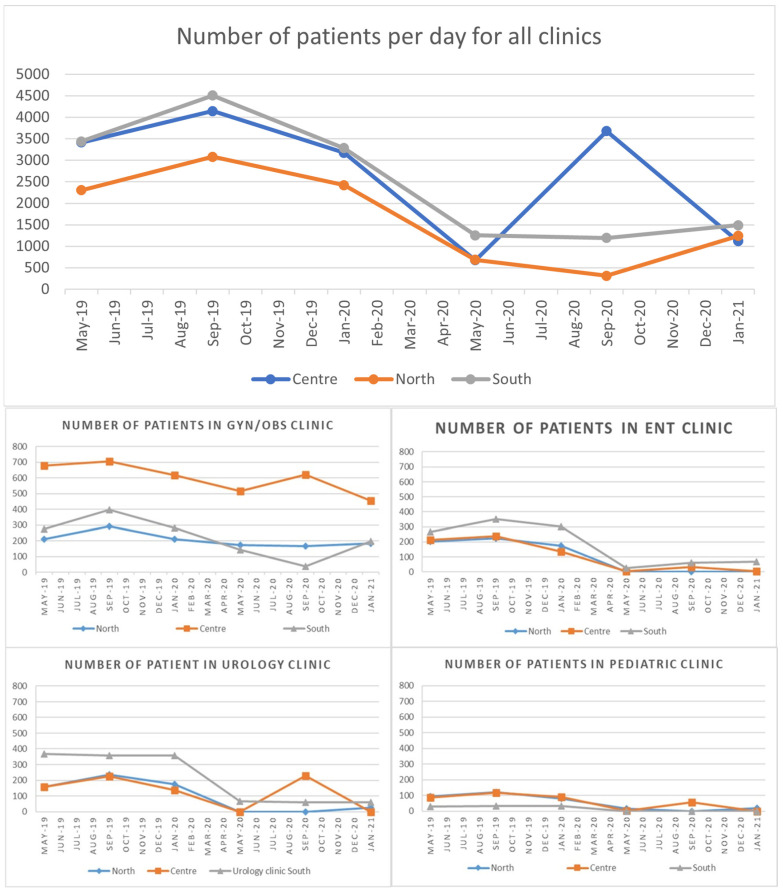
The number of patients attending all clinics and chosen different clinics in each hospital.

Throughout the epidemic, the total number of patients decreased (Figure 1). The greatest reduction occurred in the May 2020 period (compared to May 2019), with reductions of 80 percent, 70 percent, and 63 percent in the center, north, and south, respectively. In September 2020 (compared to September 2019), both the south and north hospitals maintained their downward trend, hitting 74% and 90%, respectively. The decline was 49 percent in the north, 65 percent in the center, and 55 percent in the south in January 2021 (compared to January 2020). The north and south cores had statistically significant declines.

The decrease in clinic visits had varying effects on different specializations (Figure 1). The department that was least affected was gynecology and obstetrics. In comparison, the most affected department was the Ear-Nose-Throat (ENT) department, which saw no patients in the north throughout the pandemic, no patients in the center between May 2020 and January 2021, and a major decline in the number of ENT patients in the south during the pandemic (Figure 1). Urology and pediatric surgery departments were also among the hardest hit (Figure 1).

### 3.1. Patient Age and Gender

**Figure 2:**
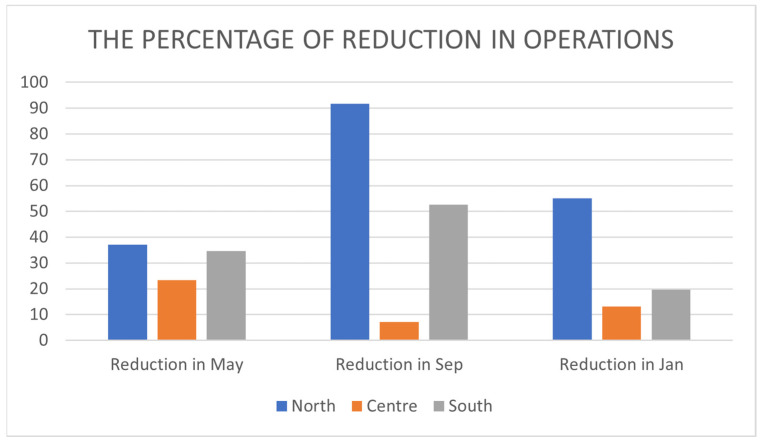
The percentage reduction in operations in each hospital.

The average age of patients in most departments remained steady; however, there was a slight but statistically insignificant increase in the average age of patients in both the oncology and ENT departments during the pandemic, compared to the pre-pandemic period (Figure 2).

Before the pandemic, the three hospitals had 60% female patients; after the pandemic, this increased to 62% (Table 2), and there was no statistically significant difference in clinic visit distribution by gender. 

### 3.2. Number, Type, and Gender Distribution of Operations

A *t*-test revealed a statistically significant difference in the number of female patients who underwent operations (decrease in the pandemic) in the north (Rafedia) and south (Alia) but not in the center (PMC), as shown in Table 2.

Table 2 reveals that a *t*-test showed a statistically significant difference in operations performed in the north (Rafedia) and south (Alia) (reduction in the pandemic) but not in the center (PMC). In comparison with the previous period, the number of northern operations declined by 37.1%, 91.8%, and 55.1% in May 2020, September 2020, and January 2021, respectively. In the center, the reductions were 23.4% (May 2020), 7.1% (September 2020), and 13% (January 2021). The south experienced 34.6 percent, 52.7 percent, and 19.6 percent reductions (Figure 2).

Major operation percentages were constant throughout the pandemic. However, small operations increased at the expense of intermediate processes. A *t*-test revealed a statistically significant difference in the type of operations performed in the north (Rafedia) and south (Alia) but not in the center (PMC), as shown in Table 2.

There was an increase in the female percentage of patients who underwent operations between January 2021 and September 2020 in the north and September 2020 in the south.

## 4. Discussion

This study found that there was a decrease in the number of patients accessing outpatient clinics in Palestinian state hospitals during the COVID-19 pandemic. The decline was greater among ENT patients, while the pandemic had little effect on the number of obstetric patients.

The difference in the number of patients admitted to public hospitals can be attributed to institutional, environmental, and individual factors. The decrease in outpatient clinic visits in Palestinian governmental hospitals was comparable in the north and south (both did not open their clinics in September 2020). However, there were notable distinctions in the central hospital, primarily because this hospital has semi-independent governance. Additionally, the hospital in the West Bank’s central district reopened outpatient clinics in June 2020 and has made significant efforts to compensate for the first lockdown, such as through doctors directly contacting patients on the phone to arrange their visits (which was not carried out before), as well as extra hours for renal, cardiac, oncology, and other clinics to serve a higher number of patients without crowding [18]. Thus, the number of patients increased in September 2020 but did not reach the levels seen in September 2019. As a result, more people have accumulated on waiting lists, jeopardizing outcomes [19].

The disparities in these patterns, particularly in September 2020 in the center, support the notion that various COVID-19 pandemic management systems would have a lesser effect on patients. This study implies that decentralizing decision-making would be beneficial and could result in improved outcomes, as shown in India. Decentralizing health services and decisions in India under COVID-19 resulted in more accurate metrics than when they were centralized [20]. Thus, the increase in cases in one Palestinian governorate should have no effect on the residents of other, less affected governorates. Additionally, in the United States of America, a scoring system was implemented to determine which operations to perform in hospitals. Each hospital was granted independence to select whether to operate, manage, or reallocate its resources based on real-time reviews. According to real-time evaluations, each hospital’s independence was given to determine when to operate, manage, or shift its resources [8]. 

To mitigate the pandemic’s direct and indirect effects, Palestinian leaders should isolate COVID-19 management facilities from major hospitals. In Australia, facilities devoted to COVID-19 were designated, alleviating the fear of hospitalization and emergency services. At the start of the pandemic, it was widely recognized that major hospitals with the best equipment and people would serve as COVID-19 management centers. After progressing through the pandemic and gaining expertise in managing the COVID-19 pandemic, it may be prudent to segregate COVID-19 management from the primary centers in order to minimize the effect on non-COVID-19 patients. The reopening of outpatient clinics in January 2021 at Alia hospital demonstrated that the number of patients has not returned to normal, despite the anticipated increase in attendance following the relocation of the COVID-19 patients to another health facility. Reports from Italy, Brazil, and the United Kingdom indicate that patients’ unwillingness to visit hospitals, administrative factors, such as staff relocation to other facilities, and financial factors such as a lack of disposables and other equipment, were among the anticipated causes [11]. Thus, an international strategy for combating any future pandemic should be developed with consideration for non-pandemic patients and a balance of efforts and resources to ensure their optimal distribution for all patients.

In January 2021, the West Bank was engulfed in a COVID-19 wave. As a result, COVID-19 had a high infection rate and caused a large number of deaths. As a further result, patients avoided hospitals, as was the case in other countries such as Pakistan, which reported a 74.5 percent decrease in attendance at healthcare facilities due to fear of COVID-19 infection [16] and also because the center suffered from the partial closure of clinics. As a result, the number of patients visiting clinics was cut in half [18].

It is critical to increase communication between the many components of Palestine’s healthcare system and the general population. In January 2021, inaccurate information exacerbated the pandemic’s effect on hospitalized patients, which may have been avoided with unambiguous warnings from authorities. A transparent system based on trustworthy, clear, concise, and targeted messaging would aid in the dissemination of accurate information and prevent incorrect information from negatively affecting people’s lives. Communication should be planned for and used appropriately in these instances.

The maintaining of near-normal gynecological and obstetric services in Palestine is a success story. Italy experienced a 50.2 percent decrease in attendance in obstetric and gynecological departments during the COVID-19 epidemic [21]. Even after the outpatient clinics were converted to COVID-19 facilities, the system continued to serve Palestinian women. According to the United Nations Population Fund, Palestine has the region’s highest rate of prenatal coverage, at 99.5 percent. Antenatal care was suspended in primary health clinics but continued as usual in hospitals during the COVID-19 crisis [22,23].

In contrast to Obs/Gyn, the three central departments most affected were ENT, urology, and pediatric surgery. This drop may be because physicians believed their patients were not emergency cases and could wait. Another possibility is that they felt there was a great danger of contracting COVID-19 infection due to their close contact with patients’ mucous droplets [24]. Thus, the ENT department could be considered an example of a setting in which doctors were the primary source of patient influence. This occurred in Italy, where 90% of ENT services were discontinued for the same reason [24]. Urologist patient reductions took a different path, owing primarily to the closure of outpatient clinics and the relocation of urology physicians to assist with the COVID crisis. The same thing occurred in New York urology departments, with very comparable reasons and outcomes [25].

Pediatric surgical patients were the most vulnerable. Pediatric surgery operations could not be postponed indefinitely. The private sector’s operating costs are considerable, and there is no state coverage referral for patients who do not frequent outpatient clinics. Reducing the number of patients examined in the clinic would add to the department’s already lengthy surgery lists due to a shortage of pediatric surgeons [26]. A Canadian study showed that the direct negative effect of COVID-19 on pediatrics was limited, but the indirect effect due to management delay, mainly due to lack of urgency, was significant and reached an up to 50% reduction in children attending healthcare services [1,27].

Each hospital saw a decrease in the number of operations. The most dramatic decline occurred in the south and north during the September 2020 period, owing to a longer closure period. In the center, the hospital opened in September 2020, but human resources for COVID-19 departments were reallocated. The American College of Surgeons postponed procedures deemed urgent or overwhelming in order to reallocate resources to COVID-19 patient management. Simultaneously, the national health service (NHS) purchased multiple beds from independent hospitals and constructed numerous field hospitals staffed by NHS employees who had been transferred from their hospitals [9].

The percentage and quantity of intermediate operations rose during the epidemic period in the center. The percentage increase was primarily attributable to a decrease in minor operations (minor operations were closed) and a fairly constant percentage of major operations. Intermediate operations, which typically do not necessitate preoperative hospital admission and whose patients do not require Intensive Care Unit care after surgery and can be quickly discharged, increased in absolute number compared to previous periods due to the preference for intermediate operations over major ones so more resources could be allocated to the management of COVID-19 patients. Worldwide, operations were been prioritized to address COVID-19 patients at the expense of non-COVID-19 patients [28,29].

This study highlighted Palestinian communities’ elective procedure cancellations. However, a concurrent study found that over 50% of Palestinians who had elective procedures cancelled during COVID-19 suffered psychological effects, while approximately 70% of the elderly suffered physical effects [30].

## 5. Conclusions

The presence of COVID-19 had an effect on the number of patients seeking outpatient care.

While some services were affected by the epidemic, hospitals in the West Bank were able to provide normal obstetric and gynecological services and assist patients requiring primary or intermediate surgery. Additionally, the system facilitated the development of context-sensitive policies that were attentive to patient requirements and hospital capacities.

## 6. Study Strengths and Limitations

This study provides a better understanding of how the Palestinian healthcare system functioned during the COVID-19 period. It is one of few studies reporting about this issue in the Middle East region.

The study is based on secondary data with an acceptable quality. However, some records had some missing information.

Using record data without patients’ consent for research could be considered unethical in some countries.

## Figures and Tables

**Table 1:** Information on the hospitals comprising the study settings adapted from Palestinian Ministry of Health, Annexes Report 2022–2021 [16].

Name of hospital	Rafedia	Palestine Medical Complex	Alia
Location	North	Center	South
Number of beds	201	279	252
Allocated COVID-19 beds during waves	50	118	98
Number of beds in the COVID-19 hospital in the same governorate	66	26	77
Date or outpatient clinic reopening	October 2020	June 2020, partial closure in September 2020–March 2021	October 2020

**Table 2:** T-test: the number, age, and gender distribution of clinic visits, and number, type, and gender distribution of operations before and after the pandemic.

Hospital		Rafedia			PMC			Alia		
Indicator	Period	Mean	Std. Deviation	*p*-Value *	Mean	Std. Deviation	*p*-Value *	Mean	Std. Deviation	*p*-Value *
No. of operations per period	Pandemic periods	132.3	93.19	0.017	316.0	88.50	0.487	214.7	26.08	0.017
	Previous periods	346.3	10.69		366.3	71.70		342.0	50.03	
	Total	239.3	131.37		341.2	77.14		278.3	78.34	
Type of operations per period	Pandemic periods	107.3	76.79	0.020	232.0	61.49	0.307	181.3	26.31	0.026
	Previous periods	278.0	18.19		282.7	42.91		294.3	50.16	
	Total	192.7	105.97		257.3	54.95		237.8	71.51	
No. of female patients in operations	Pandemic periods	84.3	64.81	0.030	167.7	37.75	0.208	122.7	5.13	0.012
	Previous periods	210.3	12.70		212.3	35.23		189.0	25.53	
	Total	147.3	80.67		190.0	40.81		155.8	39.89	
No. of patients per day in all outpatient clinics	Pandemic periods	745.7	466.37	0.007	1823.3	1620.53	0.148	1312.7	156.87	0.004
	Previous periods	2603.7	418.50		3578.0	503.45		3742.3	665.88	
	Total	1674.7	1092.11		2700.7	1440.66		2527.5	1399.35	
No. of female patients in all clinics	Pandemic periods	62.7	10.65	0.154	72.8	13.74	0.229	53.9	4.21	0.642
	Previous periods	51.8	1.33		61.5	0.53		55.1	0.21	
	Total	57.3	9.04		67.2	10.66		54.5	2.75	
Average age of patients in all clinics	Pandemic periods	37.8	1.33	0.352	34.1	2.33	0.049	38.0	3.06	0.227
	Previous periods	38.7	0.38		37.9	0.56		35.4	0.57	
	Total	38.3	0.99		36.0	2.60		36.7	2.42	

* *p*-value < 0.05 is significant.

## Data Availability

All data generated or analyzed during this study are included in this published article.
